# Plasma Cell Gingivitis: A Clinicopathological Case Report Highlighting a Systematic Diagnostic Approach and Step-by-Step Surgical Management

**DOI:** 10.7759/cureus.114714

**Published:** 2026-08-18

**Authors:** Shivangi Singh, Madhvika Patidar, Madhu S Ratre, Neha Choudhary

**Affiliations:** 1 Periodontology, Government College of Dentistry, Indore, IND; 2 Oral and Maxillofacial Pathology and Microbiology, Government College of Dentistry, Indore, IND

**Keywords:** gingival enlargement, gingivectomy, gingivitis, immunohistochemistry, plasma cells

## Abstract

Plasma cell gingivitis (PCG) is a rare benign inflammatory condition characterized by diffuse erythematous gingival enlargement with dense plasma cell infiltration. Its clinical presentation overlaps with inflammatory, autoimmune, hematological, and plasma cell proliferative disorders, making accurate diagnosis challenging and necessitating a systematic clinicopathological evaluation. This report describes a 23-year-old male who presented with persistent gingival enlargement and bleeding involving the maxillary and mandibular anterior regions for two years despite repeated nonsurgical periodontal therapy and withdrawal of a suspected allergen. Clinical examination, hematological investigations, histopathological analysis, and immunohistochemical evaluation were performed to establish the diagnosis while excluding other plasma cell-rich lesions. Histopathological examination demonstrated a dense plasma cell-rich infiltrate, while immunohistochemical evaluation using CD19 supported a reactive plasma cell-rich lesion in correlation with the clinicopathological findings. Owing to the persistence of the lesion, external bevel gingivectomy was performed, followed by diode laser-assisted recontouring and placement of a periodontal dressing. The report provides step-by-step photographic documentation of the surgical procedure. Healing was uneventful, with restoration of physiologic gingival architecture and no recurrence during one year of follow-up. This case highlights the importance of a systematic diagnostic approach integrating clinical, hematological, histopathological, and immunohistochemical findings for the accurate diagnosis of PCG. It also demonstrates the step-by-step surgical management of persistent PCG and may serve as a practical guide for clinicians managing lesions refractory to conservative therapy.

## Introduction

Plasma cell gingivitis (PCG) is an uncommon benign inflammatory condition characterized by diffuse erythematous enlargement of the gingiva associated with dense subepithelial infiltration of mature plasma cells. Plasma cell-rich mucocutaneous lesions were first described by Zoon in 1952 in the dermatologic literature [1], whereas PCG was later recognized as a distinct clinicopathological entity involving the oral cavity. The diagnosis of PCG remains challenging because its clinical presentation closely resembles that of several inflammatory, autoimmune, and hematological disorders [2]. Over time, PCG has been described in the literature by several synonymous terms, including plasma cell gingivostomatitis, allergic gingivostomatitis, and plasmacytosis of the gingiva [3].

Although the precise pathogenesis of PCG has not been fully elucidated, it is widely regarded as a hypersensitivity reaction triggered by an exogenous allergen. A variety of potential triggering factors have been reported, including cinnamon-containing products, chewing gums, mint flavoring agents, spices, herbal toothpastes [4,5], and khat leaves [6]. However, in a substantial proportion of cases, the offending allergen remains unidentified despite detailed history taking and allergy testing.

The present report describes the clinicopathological diagnosis and surgical management of generalized PCG in a young adult male, highlighting the role of immunohistochemical evaluation and the favorable clinical outcome observed during a one-year follow-up period.

## Case presentation

A 23‑year‑old male patient reported to the Department of Periodontology with a chief complaint of persistent gingival enlargement accompanied by bleeding during toothbrushing in the maxillary and mandibular anterior regions for the past two years. The enlargement was insidious in onset and gradually progressed in size over time. The patient reported undergoing repeated courses of nonsurgical periodontal therapy during the preceding two years, with only transient improvement in gingival inflammation and bleeding. His medical history was non-contributory, and no history of systemic illness, medication use, tobacco consumption, or other deleterious habits was elicited. Detailed history taking revealed that the patient had been using an ayurvedic toothpaste for approximately five years before the onset of the lesion. Following dental consultation, the patient discontinued its use, after which a reduction in erythema and subjective discomfort was noted. However, the gingival enlargement persisted despite elimination of the suspected allergen and repeated oral prophylaxis. General physical examination was unremarkable.

**Figure 1:**
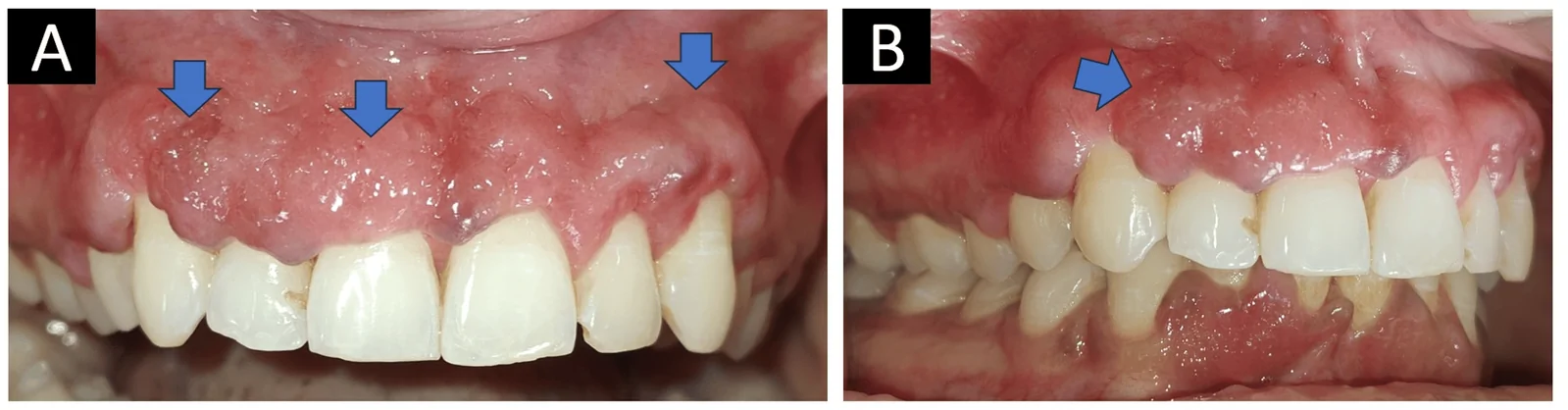
Preoperative clinical presentation. Frontal and oblique (A, B) views showing diffuse erythematous gingival enlargement involving the marginal, interdental, and attached gingiva (blue arrows).

Clinical examination revealed a diffuse enlargement involving the marginal, interdental, and attached gingiva extending from teeth #13 to #23 in the maxillary arch and #33 to #43 in the mandibular arch (Figure 1).

**Figure 2:**
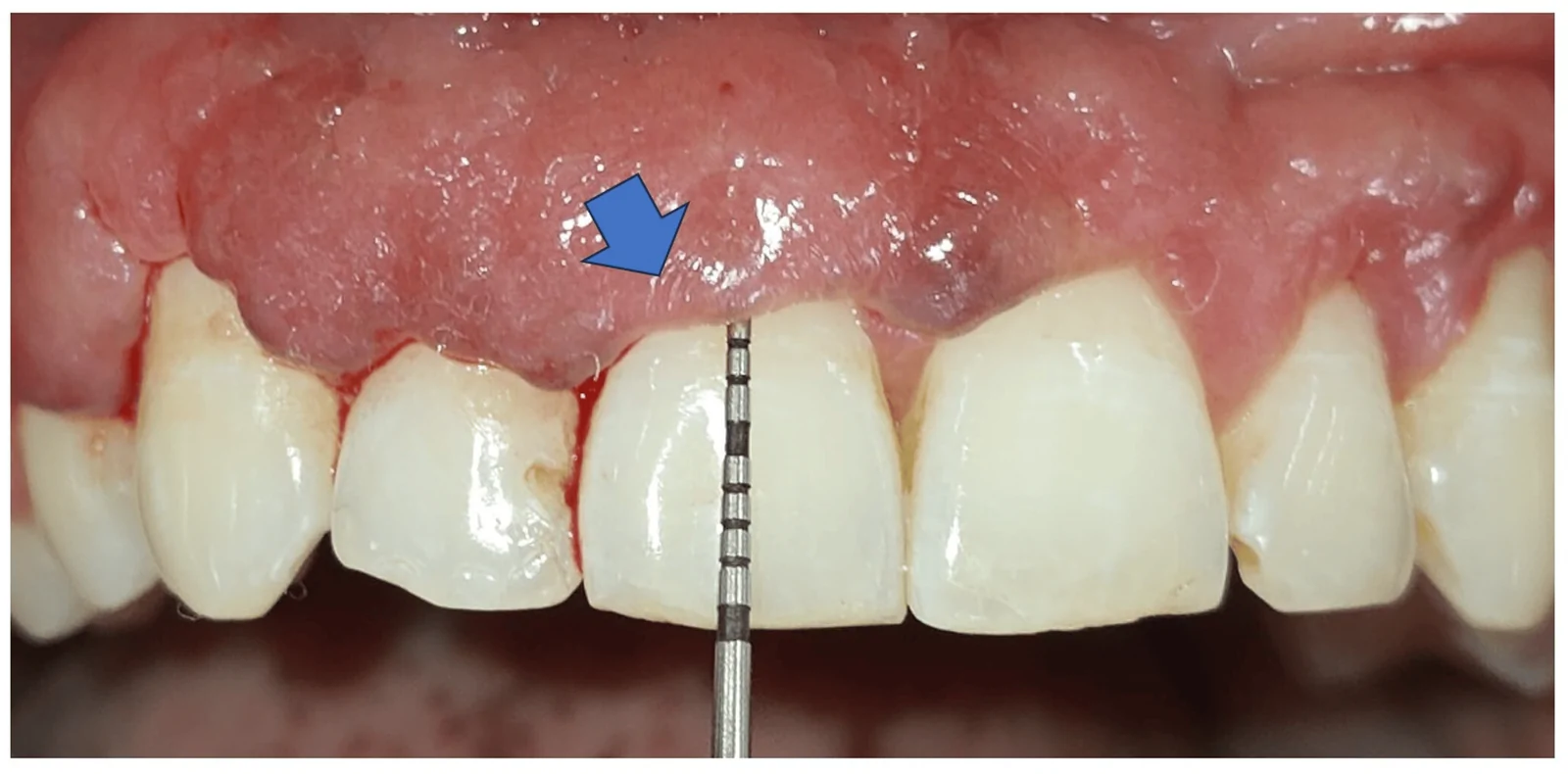
Periodontal probing demonstrating pseudopocket formation and bleeding on probing (blue arrow).

The affected gingiva appeared bright red, edematous, and friable, with a complete loss of its normal physiologic architecture. Clinical examination revealed rounded gingival margins, bulbous interdental papillae, and a smooth, glossy surface. Gentle periodontal probing elicited bleeding, and periodontal charting demonstrated generalized pseudopockets measuring 5-7 mm within the affected regions (Figure 2). 

Despite the marked inflammatory gingival enlargement, there was no evidence of clinical attachment loss, suppuration, furcation involvement, or pathological tooth mobility. Gingival changes were comparatively less pronounced in the posterior dentition. Additionally, local etiologic factors were minimal and did not correlate with the severity or extent of the gingival enlargement.

Diagnostic work-up

**Figure 3:**
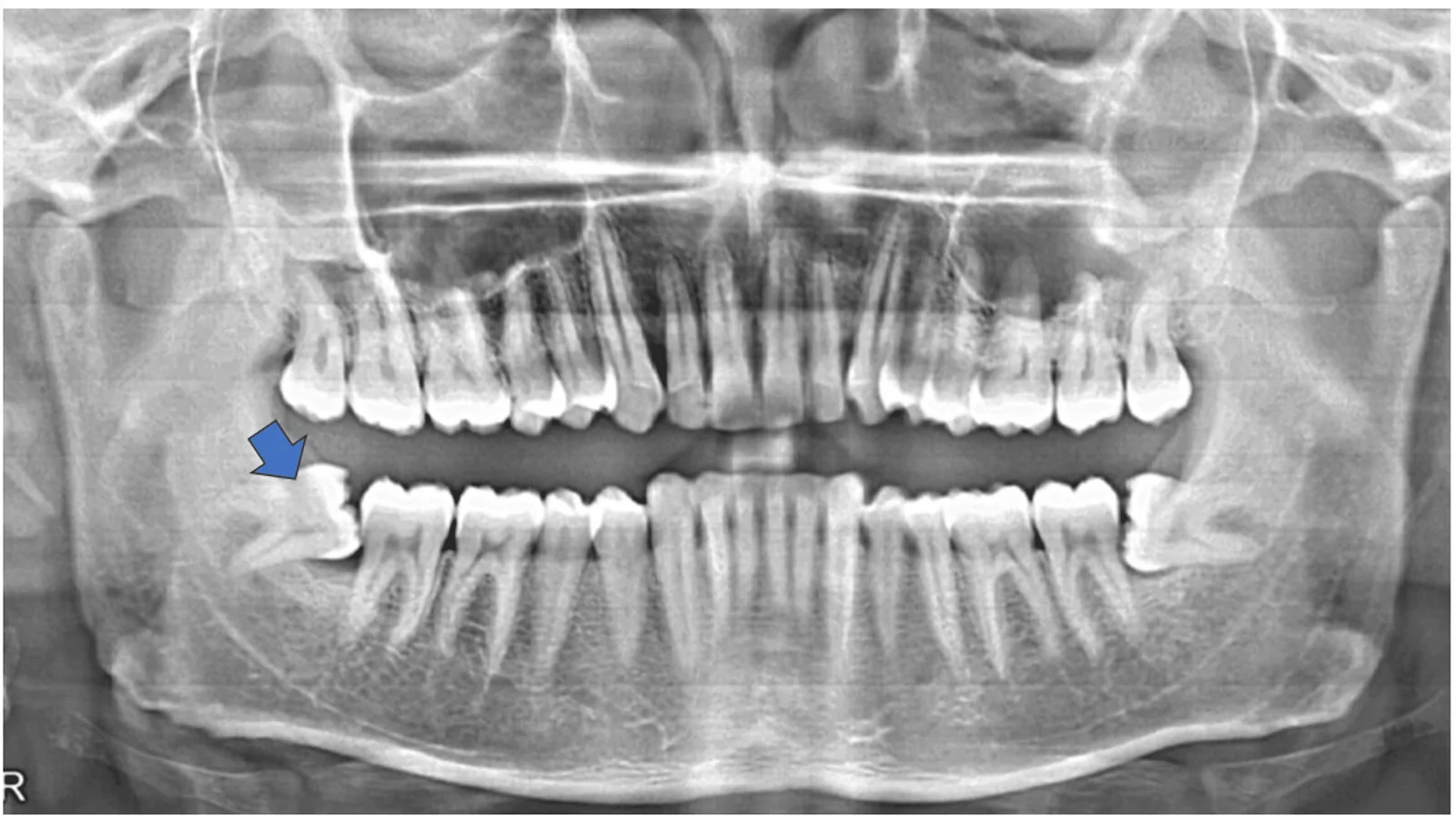
Preoperative panoramic radiograph showing preservation of alveolar bone levels without significant periodontal bone loss. Blue arrow indicates an impacted mandibular third molar (a similar incidental finding was present contralaterally).

Orthopantomographic examination demonstrated relatively normal alveolar bone levels without significant periodontal bone loss. Bilateral impacted mandibular third molars were noted incidentally (Figure 3). 

Comprehensive hematological evaluation, including a complete blood count, differential leukocyte count, hemoglobin estimation, platelet count, random blood glucose measurement, bleeding time, clotting time, and human immunodeficiency virus (HIV) serology, revealed no abnormalities.

In view of the clinical presentation, PCG was considered the provisional diagnosis, with leukemic gingival enlargement and other immune-mediated gingival disorders forming the differential diagnosis.

Initial periodontal therapy comprising supragingival and subgingival debridement, along with reinforcement of oral hygiene instructions, was carried out. At the 15-day follow-up, only minimal resolution of gingival inflammation was observed. Given the persistent nature of the lesion and its unsatisfactory response to conservative management, surgical excision of the enlarged gingival tissue was planned after obtaining informed consent from the patient.

Surgical procedure

**Figure 4:**
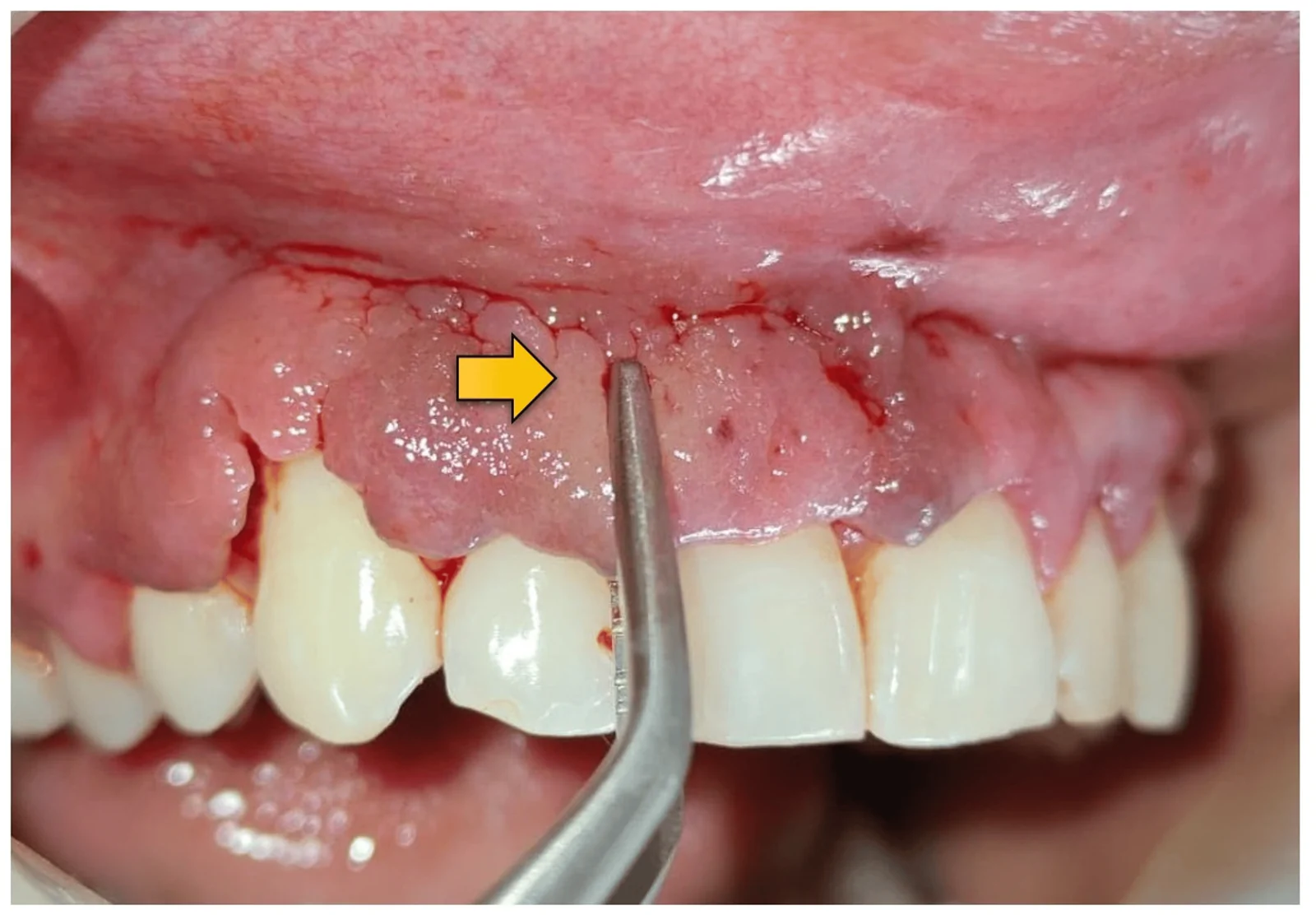
Pocket marking prior to external bevel gingivectomy. Clinical photograph showing delineation of the pseudopocket depth using a periodontal pocket marker. The yellow arrow indicates the bleeding point created by the pocket marker, which served as a guide for the external bevel gingivectomy incision.

Following administration of local anesthesia, the extent of the pseudopockets was delineated using a periodontal pocket marker (Figure 4).

**Figure 5:**
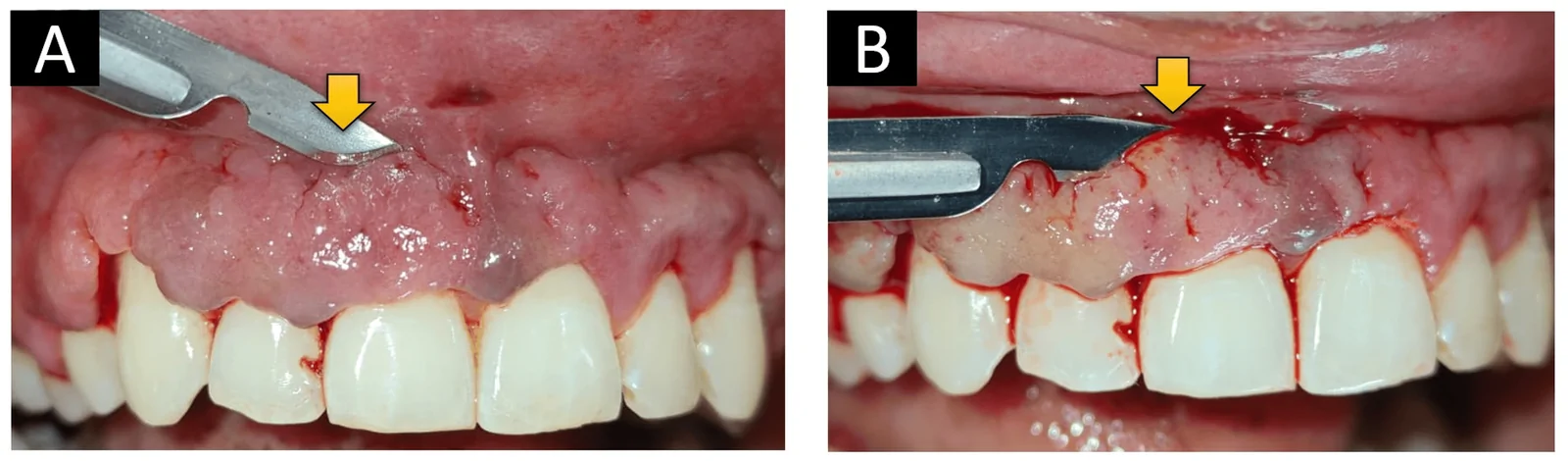
External bevel gingivectomy incision. A: placement and angulation of the No. 15C surgical blade (yellow arrow) for the external bevel incision; B: external bevel incision showing excision of the enlarged gingival tissue (yellow arrow) along the predetermined bleeding points.

An external bevel gingivectomy was performed using a No. 15C surgical blade to excise the enlarged gingival tissue and re-establish physiologic gingival contours (Figure 5).

**Figure 6:**
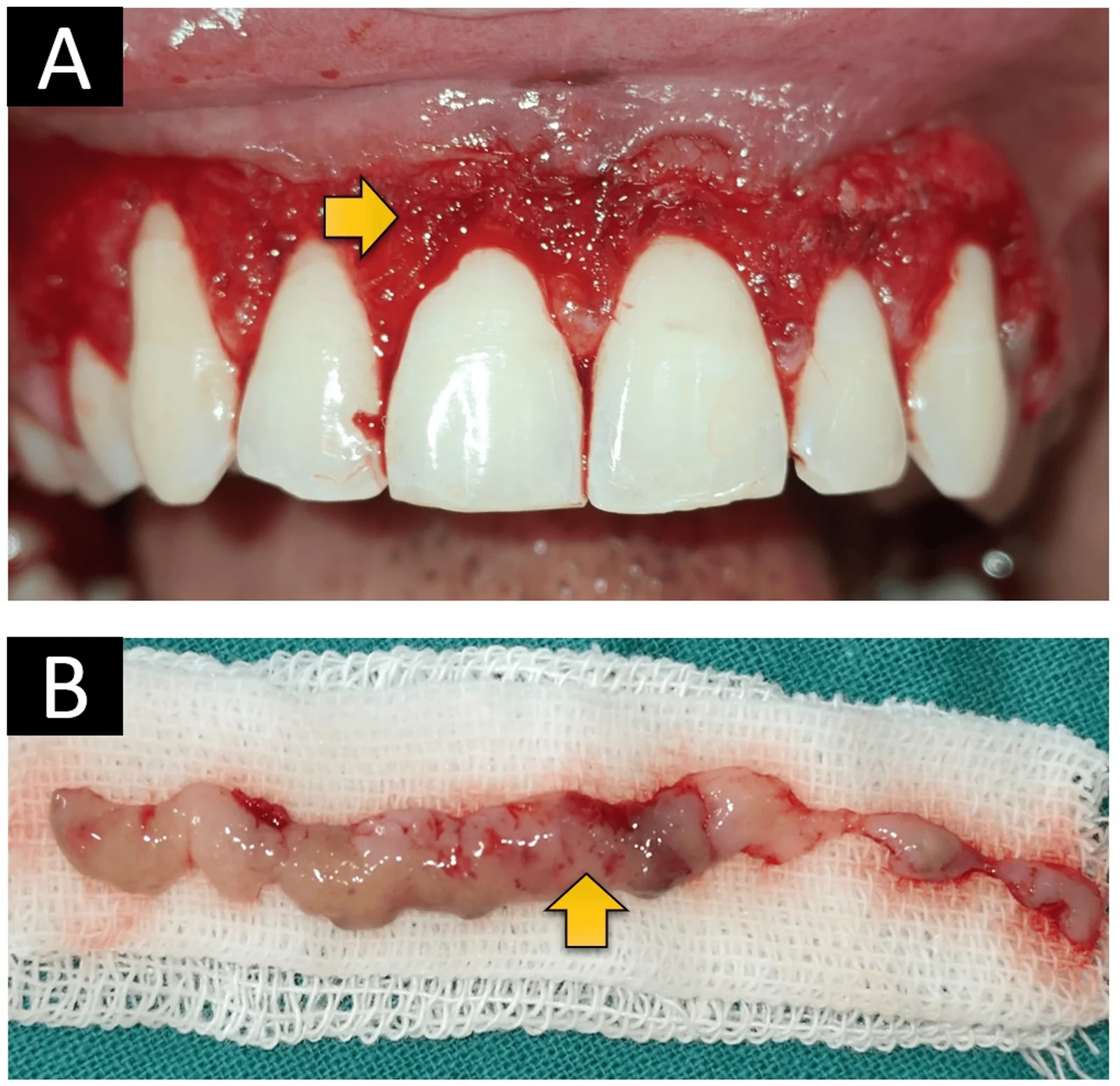
Surgical site and excised gingival tissue following external bevel gingivectomy. A: surgical site following excision showing the raw vascular surgical bed (yellow arrow); B: excised gingival tissue specimen (yellow arrow) submitted for histopathological examination.

Following excision of the enlarged gingival tissue, a raw, highly vascular surgical bed with active bleeding was evident, necessitating further hemostasis and gingival recontouring (Figure 6A). The excised tissue specimen was submitted for histopathological examination (Figure 6B).

**Figure 7:**
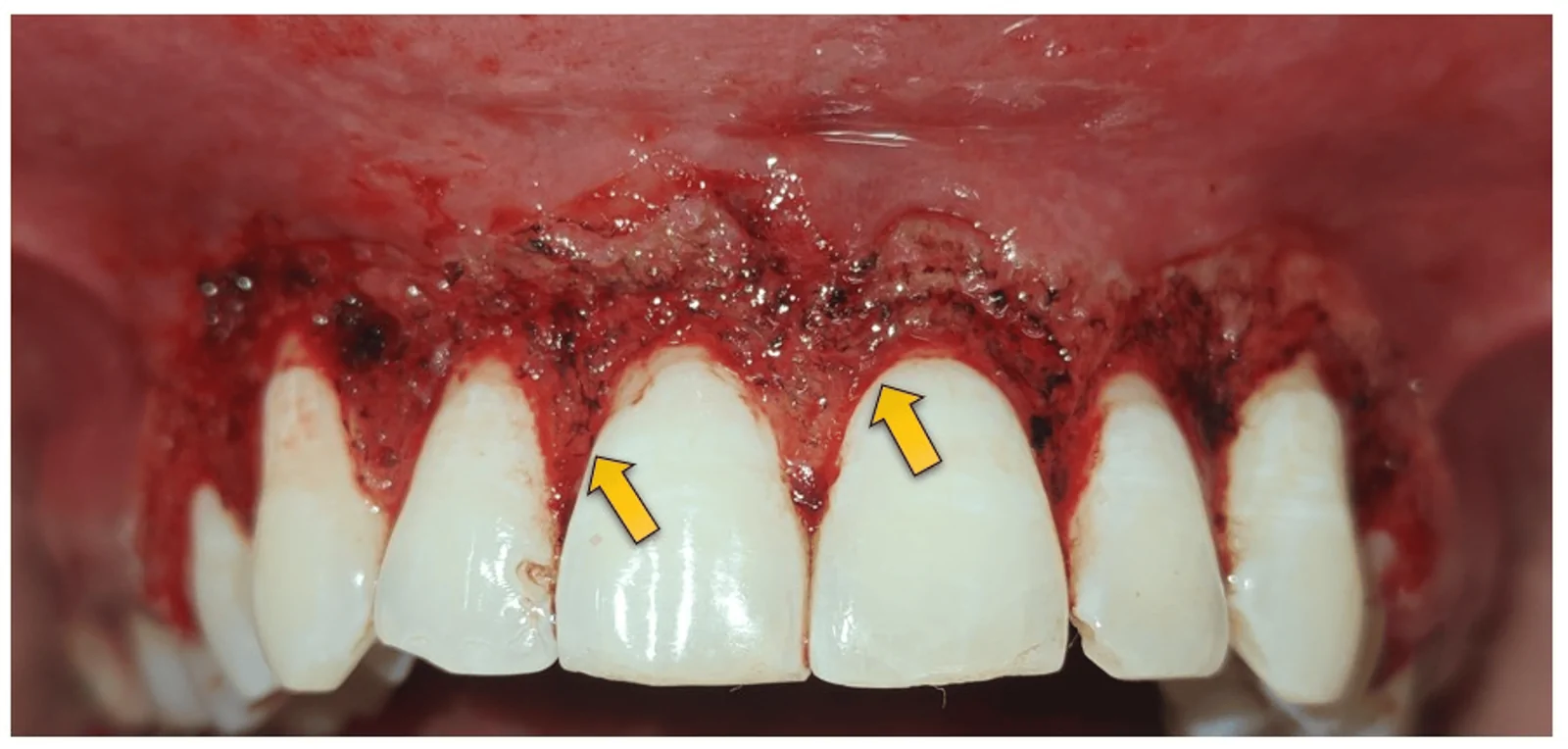
Immediate postoperative appearance following diode laser-assisted hemostasis and final gingival recontouring. Yellow arrows indicate the re-established physiologic gingival contour.

Hemostasis and additional gingival recontouring were subsequently achieved using a 980-nm diode laser in pulsed mode in a sweeping motion across the surgical field (Figure 7). 

**Figure 8:**
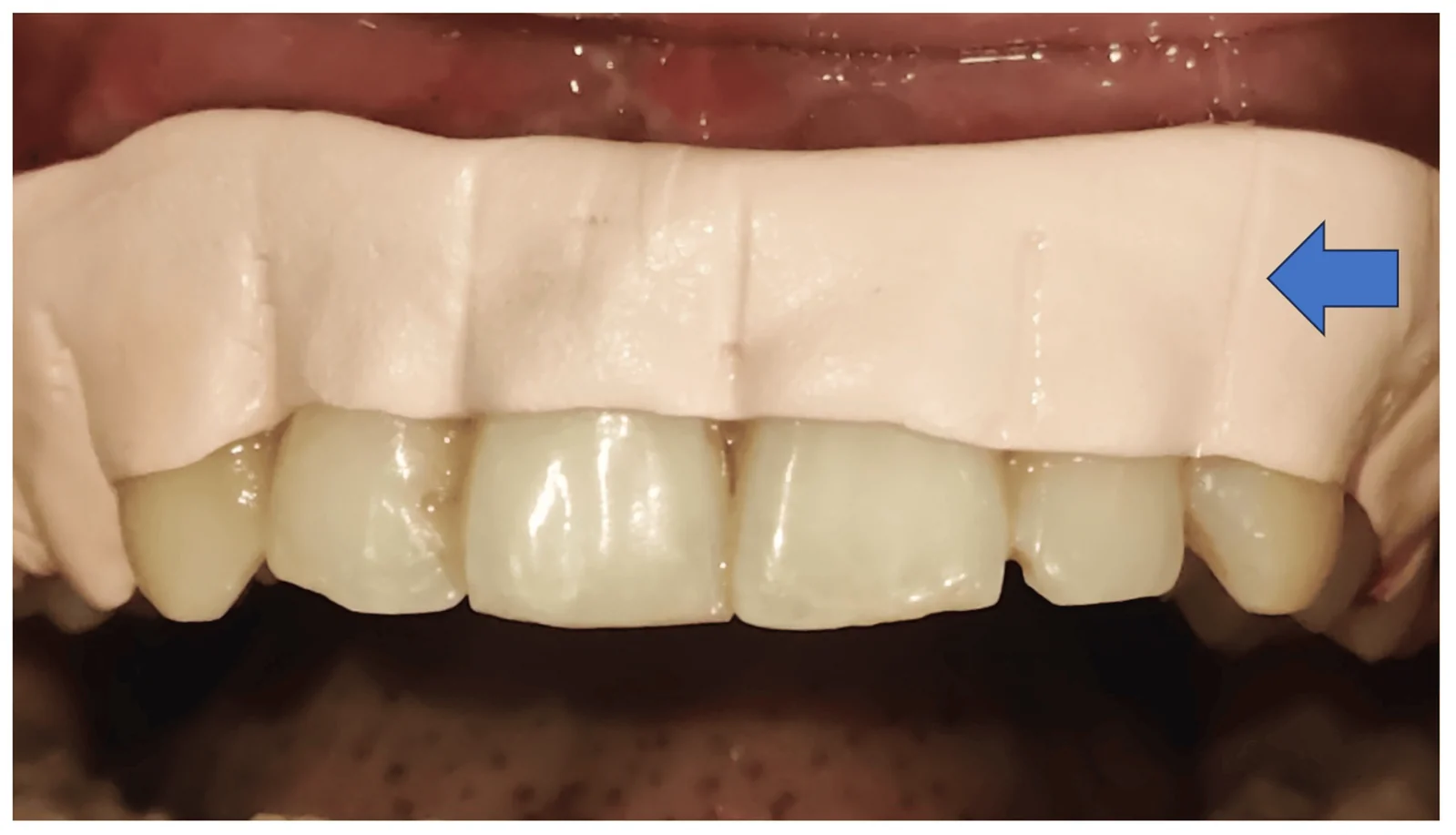
Placement of periodontal dressing following gingivectomy (blue arrow).

A periodontal dressing was placed to protect the surgical site and facilitate patient comfort during the initial healing phase (Figure 8).

Postoperatively, the patient was prescribed amoxicillin 500 mg three times daily, ibuprofen 400 mg twice daily, and a vitamin B-complex supplement for five days. Detailed postoperative instructions were provided, and the patient was scheduled for regular follow-up visits.

Histopathological features

**Figure 9:**
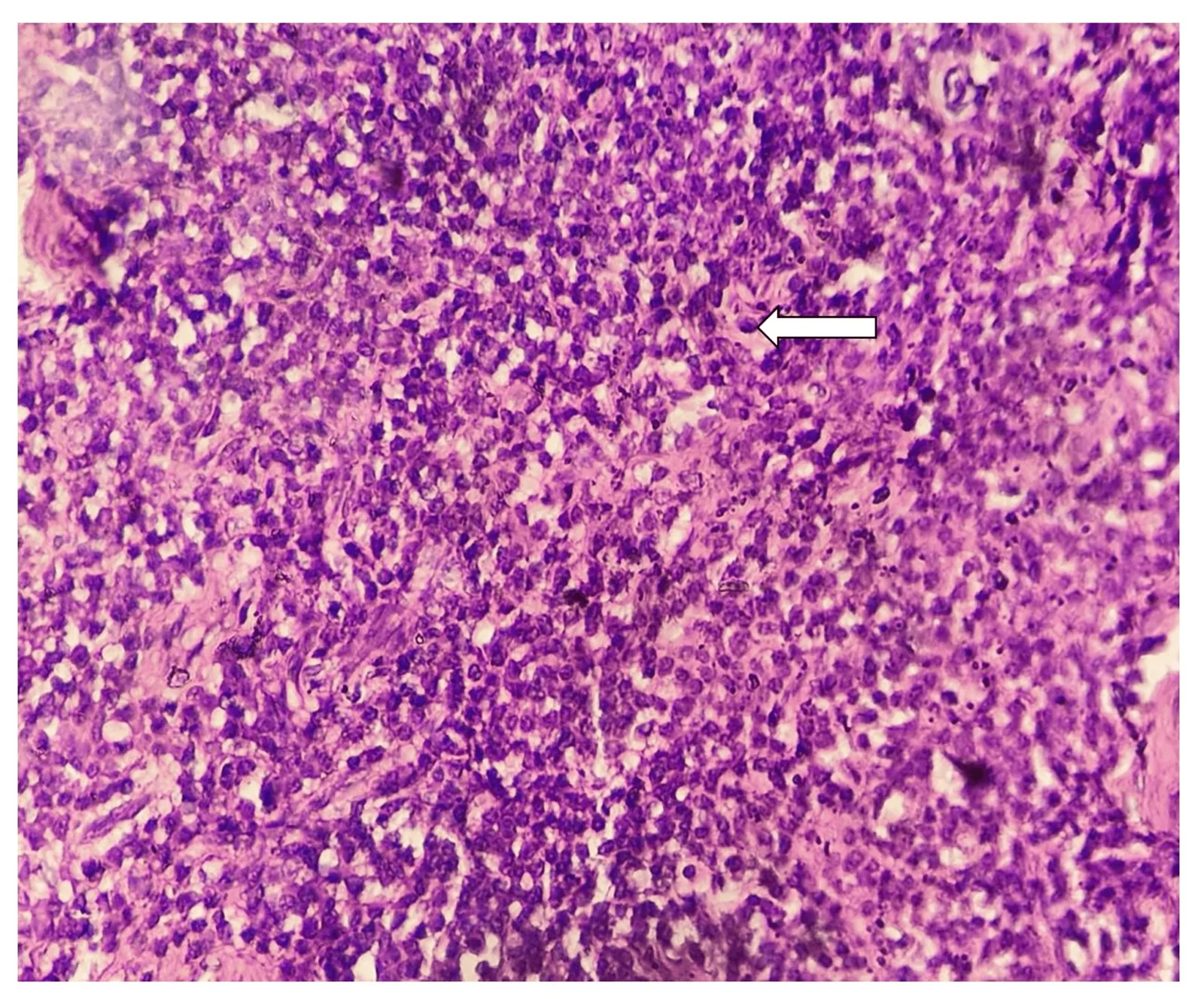
H&E (40X) showing dense subepithelial plasma cell-rich inflammatory infiltrate composed predominantly of plasma cells with eccentric nuclei (white arrow). H&E: hematoxylin and eosin staining

On histological examination, the overlying epithelium consisted of parakeratinized stratified squamous epithelium with thin rete ridges, consistent with gingival mucosa. The underlying connective tissue was highly cellular and showed a dense inflammatory infiltrate composed predominantly of plasma cells, suggestive of PCG (Figure 9). 

**Figure 10:**
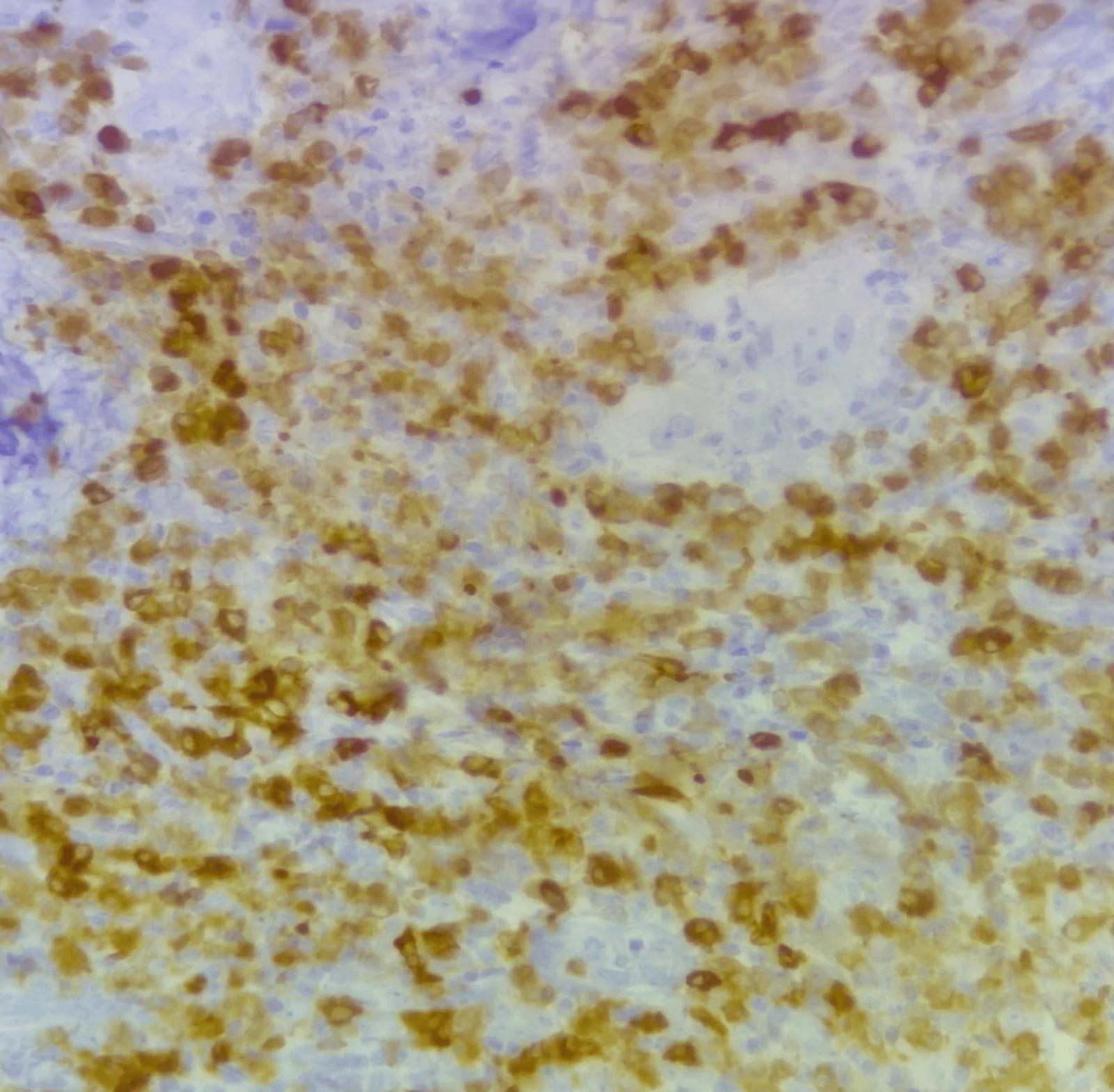
Immunohistochemistry (40X) showing CD19 positivity in the plasma cells.

A dense plasma cell-rich inflammatory infiltrate is not pathognomonic of PCG and may also be encountered in various reactive, inflammatory, and neoplastic lesions, including plasma cell mucositis, plasmacytoma, IgG4-related disease, and plasma cell dyscrasias such as multiple myeloma. Because routine histopathology alone cannot reliably distinguish a reactive polyclonal infiltrate from a monoclonal neoplasm, immunohistochemical evaluation using the CD19 marker was performed to further characterize the lesion. In this case, about 10-15% of the infiltrating cells showed strong CD19 positivity, while the remaining cells showed diffuse weaker positivity, indicating different stages of B-cell maturation and plasma cell differentiation (Figure 10).

Results

**Figure 11:**
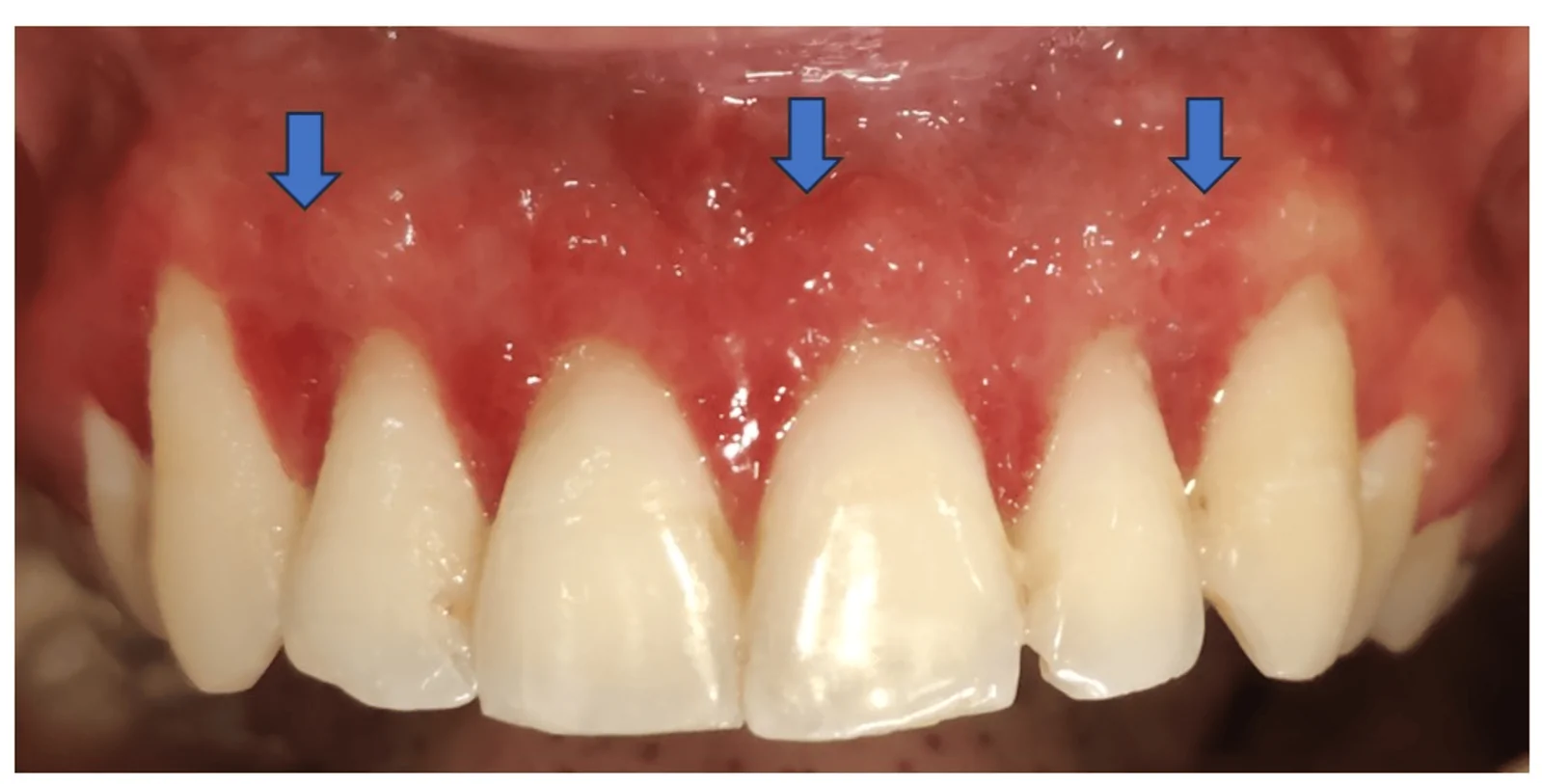
Postoperative clinical presentation after 10 days. Blue arrows indicate satisfactory epithelialization and progressive healing of the gingival tissues.

At the 10-day postoperative visit, the periodontal dressing was removed, and healing had progressed uneventfully. Clinical examination showed satisfactory epithelialization of the surgical site with a significant reduction in gingival inflammation and enlargement (Figure 11).

**Figure 12:**
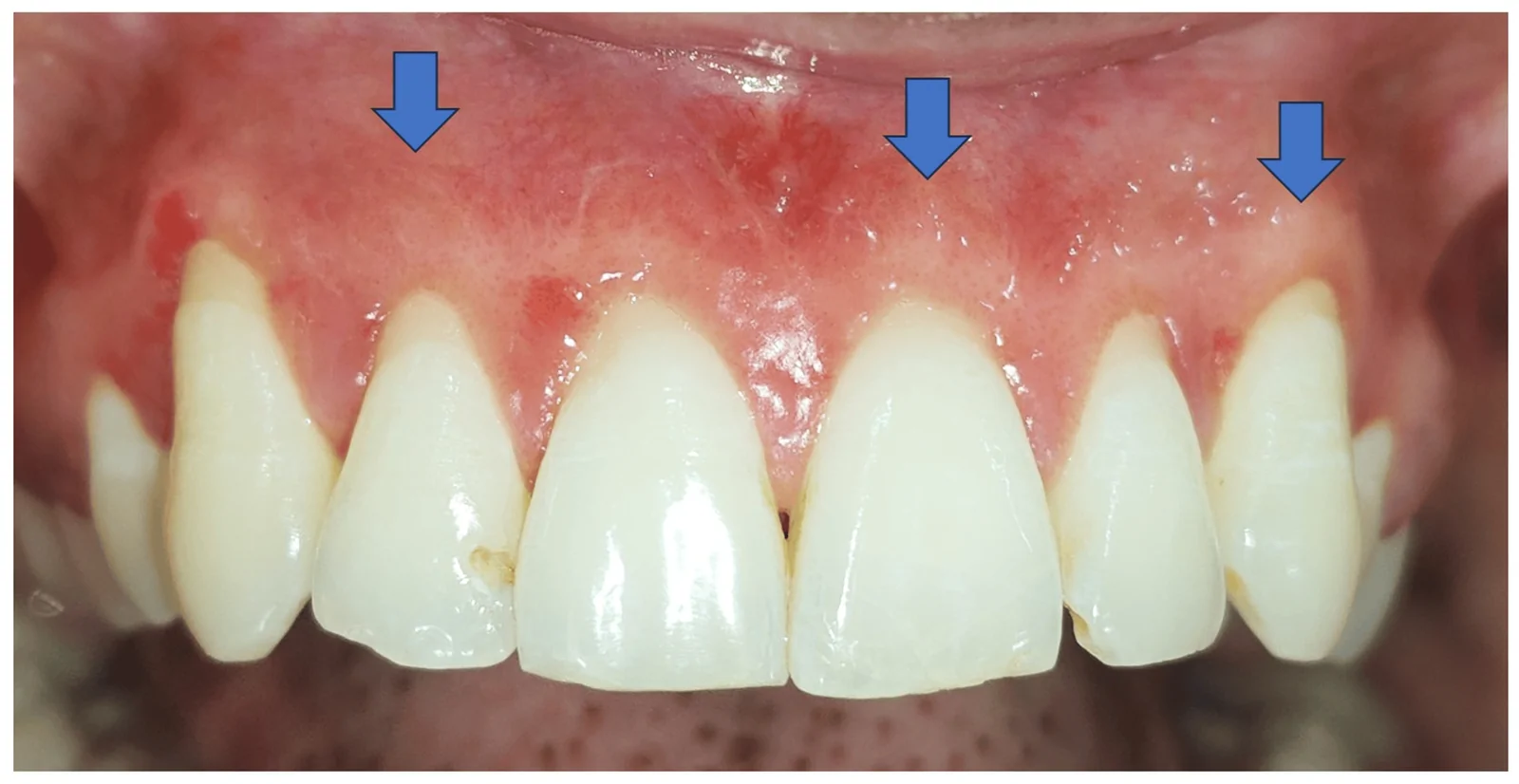
One-month postoperative clinical presentation. Blue arrows indicate progressive maturation of the gingival tissues with restoration of physiologic gingival contour.

Progressive improvement in gingival color, contour, and consistency was observed during subsequent follow-up examinations (Figure 12). 

**Figure 13:**
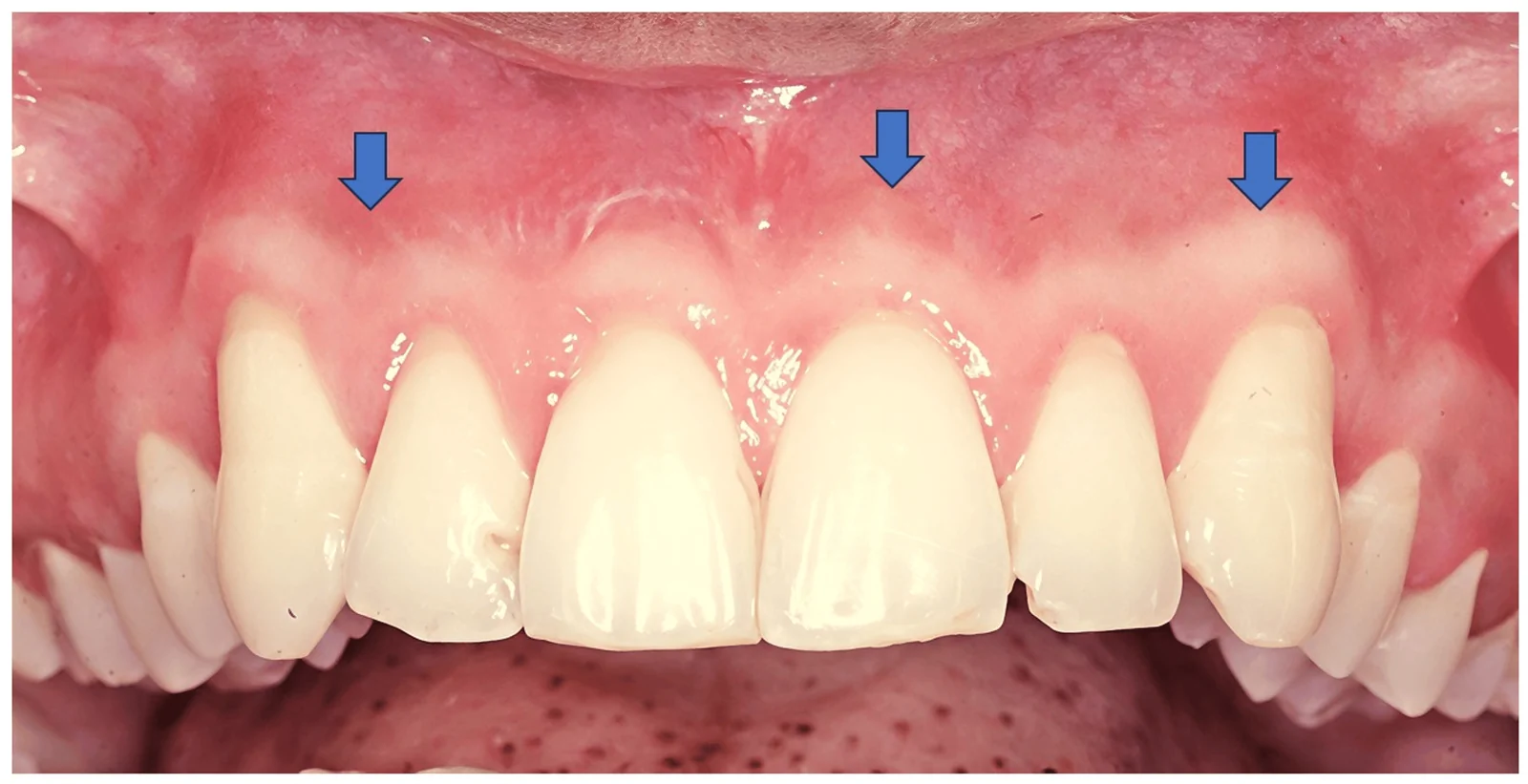
One-year postoperative clinical presentation. Blue arrows indicate restoration of physiologic gingival architecture with healthy gingival contour and absence of recurrent gingival enlargement.

At the one-year follow-up examination, the gingiva demonstrated a healthy clinical appearance with restoration of physiologic gingival architecture and absence of inflammatory signs (Figure 13). No bleeding on probing or recurrence of gingival enlargement was observed during the one-year follow-up period.

## Discussion

PCG is an uncommon benign inflammatory disorder characterized histopathologically by a dense subepithelial infiltrate composed predominantly of plasma cells. Clinically, it usually manifests as diffuse erythematous enlargement involving the marginal, interdental, and attached gingiva, frequently extending across the entire width of the keratinized gingiva [7,8].

Although the precise pathogenesis of PCG has not been fully elucidated, it is widely regarded as a hypersensitivity reaction triggered by an exogenous allergen.

In 1995, Gargiulo et al. proposed an etiological classification of PCG into three categories: allergen-induced, neoplastic, and lesions of unknown origin [9]. The present case was characterized by diffuse erythematous gingival enlargement extending to the mucogingival junction and involving almost the entire width of the attached gingiva, a clinical pattern not typically observed in conventional plaque-induced gingivitis. Despite partial resolution of the inflammatory changes after conventional periodontal therapy, the gingival enlargement remained persistent. This incomplete response was inconsistent with a purely plaque-induced etiology and prompted a comprehensive diagnostic evaluation. These findings are consistent with those reported by Joshi and Shukla, who documented persistent gingival enlargement despite initial periodontal therapy [7]. In contrast to our findings, Arduino et al. observed significant regression of gingival enlargement following phase I periodontal therapy alone [10].

Accurate diagnosis of PCG requires exclusion of several inflammatory, autoimmune, hematological, and plasma cell proliferative disorders with similar clinical and histopathological features. Histopathological examination of the excised tissue is essential for diagnosis; however, dense plasma cell infiltrates may also be encountered in chronic inflammatory periodontal lesions and plasma cell neoplasms. Clinically, PCG can closely resemble conditions such as acute leukemia, lichen planus, and mucous membrane pemphigoid. Histopathologically, it may simulate extramedullary plasmacytoma, multiple myeloma, plasma cell granuloma, and other lesions characterized by dense plasma cell infiltration [11,12].

CD19 was selected as an immunohistochemical marker in the present case because of its utility in distinguishing reactive plasmacytic infiltrates from neoplastic plasma cell proliferations. As a pan-B-cell antigen expressed throughout B-cell maturation, CD19 is generally retained, although often with diminished intensity, on normal and reactive plasma cells, whereas neoplastic plasma cells in plasma cell dyscrasias typically lack CD19 expression. In contrast, markers such as CD138 and CD38 are expressed by both reactive and neoplastic plasma cells and therefore have limited discriminatory value in this context. In the present case, approximately 10-15% of infiltrating cells demonstrated strong CD19 positivity (Figure 10), while the remainder exhibited diffuse weak to moderate expression, a pattern consistent with heterogeneous B-cell maturation and reactive plasma cell differentiation. The observed preservation of CD19 expression, interpreted in conjunction with the clinical and histopathological findings, favored a reactive plasma cell-rich process rather than an overtly neoplastic proliferation.

Accordingly, establishing a definitive diagnosis relies on comprehensive clinicopathological correlation in conjunction with appropriate immunohistochemical evaluation [13].

Recent literature has further emphasized the heterogeneous clinical spectrum and diagnostic complexity of PCG. In a multidisciplinary retrospective review of 37 cases previously identified as gingivitis with plasma cell infiltrates, Louisy et al. [14] found that seven cases were ultimately reclassified as other disorders, including oral lichen planus, plasma cell granuloma, plasmacytoma, and mucous membrane pemphigoid, while the remaining cases were classified as reactive PCG (n=18) or idiopathic PCG (n=12). These findings reinforce the importance of clinicopathological correlation and exclusion of secondary causes before establishing a diagnosis of PCG. Similarly, Fitzpatrick et al., in a 23-year retrospective analysis of 107 cases of oral plasma cell mucositis/PCG, reported a threefold increase in the proportion of biopsy specimens diagnosed with these lesions after 2020, although no statistically significant differences in age, sex, anatomical location, or histological features were observed between cases diagnosed before and after 2020 [15].

More recently, a systematic review [16] identified 19 histopathologically confirmed cases from 16 publications, with a predominance of female patients and involvement of the anterior maxilla in all included cases; treatment approaches varied from elimination of suspected etiological factors and topical therapy to lesion removal and photobiomodulation. These contemporary observations are broadly consistent with the present case, in which the marked gingival enlargement was disproportionate to local plaque-related factors, persisted despite initial periodontal therapy, and prompted a systematic diagnostic evaluation before definitive surgical management. However, the present case differed from the cases included in the recent systematic review in demonstrating extensive bilateral involvement of the anterior maxillary and mandibular gingiva, with enlargement extending across the attached gingiva to the mucogingival junction. The temporal association with prolonged use of an Ayurvedic toothpaste is compatible with the proposed role of exogenous oral products as potential triggering factors, although a specific causative allergen could not be established. The absence of recurrence during one year of follow-up following surgical management also contributes to the limited literature describing outcomes after surgical treatment of persistent PCG.

In the present case, persistence of the lesion despite withdrawal of the suspected allergen and nonsurgical periodontal therapy, together with the absence of attachment or alveolar bone loss and the presence of adequate keratinized gingiva, supported surgical management. External bevel gingivectomy was therefore performed to excise the enlarged tissue and restore physiologic gingival contours. The favorable clinical response, with complete resolution of gingival enlargement and no recurrence during one year of follow-up, supports surgical excision as a reasonable treatment option for persistent PCG refractory to conservative measures.

The present case provides sequential documentation of the diagnostic work-up and surgical management from initial presentation through one-year follow-up, thereby offering practical guidance for clinicians managing similar cases. Overall, this case emphasizes that persistent diffuse gingival enlargement disproportionate to local etiological factors warrants systematic evaluation, including hematological investigations, histopathological examination, and appropriate immunohistochemical assessment. Such an approach facilitates accurate diagnosis, exclusion of clinically similar reactive and neoplastic disorders, and appropriate treatment planning.

Limitations

This report has certain limitations inherent to its single-case design, and the findings may not be generalizable to all patients with PCG. Immunohistochemical evaluation was limited to CD19; additional markers and kappa and lambda light-chain assessment were not performed, limiting further immunophenotypic characterization and direct assessment of clonality. Although discontinuation of the suspected Ayurvedic toothpaste was followed by improvement in erythema and subjective discomfort, the specific causative allergen could not be established. Furthermore, although no recurrence was observed during the one-year follow-up period, longer-term follow-up would provide additional information regarding the durability of the treatment outcome.

## Conclusions

PCG should be considered in the differential diagnosis of persistent diffuse gingival enlargement, particularly when the clinical severity is disproportionate to local etiological factors and response to conventional periodontal therapy is inadequate. Accurate diagnosis requires correlation of clinical, hematological, histopathological, and, where indicated, immunohistochemical findings. In persistent cases unresponsive to conservative measures, surgical management may provide favorable and stable clinical outcomes, as demonstrated by the absence of recurrence during the one-year follow-up in the present case.
